# Determinants of use of supervised delivery care under Ghana’s fee exemption policy for maternal healthcare: the case of the Central Region

**DOI:** 10.1186/s12884-016-0960-6

**Published:** 2016-07-19

**Authors:** Henrietta Asante-Sarpong, Adobea Yaa Owusu, Sheela Saravanan, Ernest Appiah, Mumuni Abu

**Affiliations:** Institute of Statistical Social and Economic Research, University of Ghana, P.O. Box LG 74, Legon, Accra Ghana; South Asia Institute, Im Neuenheimer Feld 330, 69120 Heidelberg, Germany; Regional Institute for Population Studies, University of Ghana, P.O. Box LG 96, Legon, Accra Ghana

**Keywords:** Maternal deaths, Supervised care, Fee exemption policy for maternity care, Maternal healthcare

## Abstract

**Background:**

Improving access to supervised and emergency obstetric care resources through fee reduction/exemption maternity care initiatives has been touted as one major strategy to avoiding preventable maternal deaths. Evaluations on the effect of Ghana’s fee exemption policy for maternal healthcare have largely focused on how it has influenced health outcomes and patterns of use of supervised care with little attention to understanding the main factors influencing use. This study therefore sought to explore the main individual and health system factors influencing use of delivery care services under the policy initiative in the Central Region.

**Methods:**

A cross-sectional study was conducted using 412 mothers with children aged less than one year in one largely rural and another largely urban districts in the Central Region of Ghana from September to December 2013. Data were collected using a questionnaire survey on the socio-demographic characteristics of mothers, their knowledge and use of care under the fee free policy. Chi-square and Binary Logistic Regression tests were used to evaluate the main determinants of delivery care use under the policy.

**Results:**

Out of the 412 mothers interviewed, 268 (65 %) reported having delivered their most recent birth under the fee exemption policy even though awareness about the policy was almost universal 401 (97.3 %) among respondents. Utilization however differed for the two study districts. Respondents in the Cape Coast Metropolis (largely urban) used delivery service more (75.7 %) than those in the largely rural Assin North Municipal area (54.4 %).

Binary logistic regression results identified maternal age, parity, religion, place of residence, awareness and knowledge about the fee exemption policy for maternal healthcare as significantly associated with the likelihood of delivery care use under the policy. The likelihood of using supervised delivery care under the policy was lower for mothers aged 20–29 compared to those in the age bracket of 40–49 (Odds ratio (OR) = 0.069, *p* = 0.003). For their index (last child), mothers who already had 1, 2 or 3 births were more likely to deliver under the policy than those with five or more births.

Mothers living in urban areas were 3.79 times more likely to use delivery services under the policy than those living in rural areas (OR = 3.793, *p* = 0.000). The likelihood of using delivery services under the policy was higher for mothers who were aware and had full knowledge of the total benefit package of the policy (OR = 13.820, *p* = 0.022 and OR = 2.985, *p* = 0.001 for awareness and full knowledge respectively).

**Conclusions:**

Delivery service use under the free maternal healthcare policy is relatively low (65 %) when compared with nearly universal awareness (97.3 %) about the policy. Factors influencing delivery service use under the policy operate at both individual and policy implementation levels. Effective interventions to improve delivery service use under the policy should target the underlying individual and health policy implementation factors identified in the study.

## Background

Maternal mortality is a major public health problem globally but most especially in sub-Saharan Africa [1]. In Ghana, maternal mortality continues to be pervasive and improvements have been rather slow. The country’s Maternal Mortality Ratio (MMR) is estimated to be 350 per 100,000 live births [1]. Investments in supervised and emergency obstetric care resources have been noted as being critical to avoiding these deaths as most deaths, occur around delivery and are caused by haemorrhage [2].

Multiple challenges however confront women’s quest to access supervised care. These challenges include poor or inadequate health infrastructure, limited or unavailable medical supplies [3], adherence to negative socio-cultural beliefs and practices about pregnancy and child birth [4] and bad or non-existent transportation infrastructure to the nearest health facilities [5]. Financial challenges to accessing care have been particularly highlighted as critical to the use of supervised care [6, 7].

To address the financial challenges to accessing supervised care at birth, several counties including Ghana have adopted innovative financing mechanisms to address financial challenges to seeking care. The mechanisms introduced include fee exemptions, cash assistance, voluntary service contributions and public-private partnerships [8].

Studies on the effect of these financing mechanisms in low-and middle-income countries have focused on demand for obstetric services and healthcare expenditures. Ir, Souk and Van Damme [9] assessed the effectiveness of a Voucher Scheme introduced by the Cambodian Government in 2007 to improve access to skilled attendance for poor women in three rural health districts. The authors noted that even though the scheme had strong potential for reducing financial barriers to accessing skilled care at birth, other interventions for improving the supply of sufficient quality maternity services was necessary for the scheme to achieve its full potential [9]. In Burkina Faso Ridde, Kouanda, Bado, Bado & Haddad [10] assessed the effect of a national maternal healthcare subsidy policy on household spending on facility-based deliveries and found that the policy was very effective in reducing household costs for delivery care [10]. Ridde and Diarra [11] have also expressed the need for further attention to be given to sustainable financing mechanisms for the effective implementation of fee-exemption initiatives [11].

In 2003, the government of Ghana introduced a delivery fee exemption policy that exempts all pregnant women from paying for delivery care in public, mission and some private facilities. Otherwise known as the “free delivery policy”, the policy exempts all pregnant women from paying for all normal deliveries and the management of all assisted deliveries including caesarean sections. Under the policy, women are also exempted from paying for the management of medical and surgical complications arising out of deliveries, including the repair of vesico-vaginal and recto-vaginal fistulae [12].

Similar to other studies that have been undertaken in low- and middle- income countries on maternal healthcare financing mechanisms, previous studies in Ghana have largely examined the delivery fee exemption policy in terms of how it has influenced utilisation patterns [13, 14] and healthcare expenditures [15]. Less attention has been given to understanding the driving factors influencing use within local contexts. This study therefore sought to explore the key individual and health policy implementation factors influencing use of delivery care services under Ghana’s free delivery policy.

## Methods

### Study setting

The Central Region was selected for the study based on the following reasons: (i) the region was selected among the first four pilot regions in which the fee exemption policy for maternal deliveries was implemented in 2003. (ii) compared to the three other pilot regions (Northern, Upper East and Upper West), the Central Region has not witnessed improvements in skilled attendance rate particularly between 2008 and 2012 when services under the policy was administered through the NHIS. (iii) Contrastingly the region also has a Maternal Mortality Ratio of 520/100,000 live births [16] a ratio which is far higher than the national average of 350/100,000 live births [1].

Two districts (Cape Coast Metropolitan Area and Assin North Municipal Area) in the Central Region were purposively selected from the seventeen districts of the region for the study. The two districts compared to the others have the highest maternal mortality ratios [17]. Cape Coast Metro is also largely urban whereas Assin North is largely rural [16] a scenario that provides an opportunity to assess differences in care received under the policy within rural and urban settings.

### Study participants

The primary study population was mothers of reproductive age (15–49 years) with children under one year of age. The choice of women with these characteristics was based on the study goal that aims to examine factors influencing delivery service use under the ‘free maternal healthcare’ policy. The target population largely serves as the potential users and benefactors of services under the policy. Secondly, mothers whose most recent birth occurred 12 months prior to the survey are most likely to recall and give a better account of their experiences.

A total of 412 mothers were selected from the study districts (Cape Coast, *n* = 206; Assin North, *n* = 206) using a combined multi-staged, stratified and simple random sampling techniques. The sample size was determined with recourse to Kish [18] since the population under study was homogeneous and the total population of mothers with at least a child under one is not known [18]. The study participants were selected from eight different localities (4 rural and 4 urban) identified from a list of all rural and urban localities in the study areas through a simple random approach. The calculated sample populations for rural and urban areas from the total sample was shared equally across the study localities as the study population was homogeneous and therefore likely to share similar views and experiences.

This was followed by the identification of households in each locality from which women were interviewed. The identification of households began with a surveillance exercise that was undertaken by the research assistants through the help of local opinion leaders and healthcare volunteers in the respective localities. Having identified households in which mothers eligible for the interviews reside, a list/sampling frame of these mothers was produced for each locality. From the sampling frame of mothers produced for each locality, a simple random approach (writing the names of each eligible respondent on pieces of papers, shaking them arbitrarily and selecting required number from the whole) was later employed to select the total number of respondents earmarked for each settlement or locality.

### Data generation

Data were collected through the administration of a standardized questionnaire. The data collection exercise was undertaken concurrently in the two study districts between the months of September to December 2013. The questionnaire was administered to all 412 mothers and consisted of sections that asked questions related to their background characteristics such as age, marital status, education, religion and ethnicity, place of residence, employment status and parity.

Similar questions were asked for the background information of their spouses/partners. The other sections had questions related to their obstetric history, their knowledge and perceived need of services provided under the free delivery policy; and experiences with use of delivery care under the delivery fee exemption policy.

### Study variables

The dependent variable is use of delivery services under the fee exemption policy. It was derived from the question, “Did you deliver for free under the ‘free delivery policy’ or you paid for delivery services?” The dependent variable was measured by using the labels 1 and 2 with 1 being ‘Delivery for free’ and 2, ‘Delivery not for free.

The independent variables were selected with reference to what has been used in previous studies. They comprised of those related to the socio-demographic characteristics of women as well as their partners and others on the free delivery policy. The variables selected on the socio-demographic characteristics of women and their husbands/partners included age, religion, level of education, employment status, marital status, parity, place of residence and ethnicity. The variables were defined and measured as follows.

Education was defined as completed educational status and was ranked from 1 to 5 with label 1 for No formal education, 2 for primary education, 3 for Middle/Junior High School (JHS), 4 for Secondary/Senior High School (SHS)/Technical education, 5 for higher than secondary. Employment status was defined as the category of work respondents were engaged in and was ranked from 1 to 5 with 1 for ‘unemployed’, 2 (Self-employed), 3 (Paid employee), 4 (Paid informal worker) and 5 (Other forms of employment mostly seasonal employment). Parity referred to the total number of live births a woman had and was ranked from 1 to 5 with 1 for parity one, 2 for parity two, 3 for parity three, 4 for parity 4 and 5 for parity five and above. Place of residence was ranked 1 and 2 with 1 being Urban and 2, Rural. The marital status of respondents was ranked into three categories. Those who were currently married or cohabiting were assigned rank 1, formerly married, rank 2 and single, never married women given rank 3.

The variables for the free delivery policy were awareness about the ‘free delivery’ policy and knowledge about benefit package for the ‘free delivery’ policy. Awareness of the free delivery policy was defined as having heard about the existence of the policy and ranked 1 for a ‘Yes’ and 2 for a ‘No’. Knowledge about the policy was also ranked as 1 and 2 with 1 referring to answering yes to having knowledge about the full benefit package of the policy and 2 for answering no to having knowledge about the policy.

### Statistical analysis

The Statistical Package for the Social Sciences (SPSS) software version 20.0 was used to analyze the quantitative data. Descriptive statistics were used for frequency counts and percentage distribution of background characteristics of respondents as well as prevalence of use of delivery services under the free maternal healthcare policy. The Chi-Square test was used to test for the statistical associations between use of delivery care and other independent variables. The binary-logistic regression model was used for identifying the main determinants of use of delivery care under the fee exemption policy.

Three models containing variables of interest were fitted for the outcome variable (use of delivery care). The first model contained variables on the socio-demographic characteristics of mothers. This model was used to assess the association between their socio-demographic characteristics and use of delivery services. The second model contained variables on the demographic characteristics of the selected mothers and that of their husbands/partners. This helped to assess whether the husband’s/partner’s characteristics influenced the association between the background characteristics of the woman and the outcome variable. A third model containing variables on the socio-demographic characteristics of the woman as well as that of their husbands/partners and the free delivery policy was also estimated. The final model (Model 3) was used to estimate whether health policy and husbands’/partners’ socio- demographic characteristic factors moderate the association between mothers’ socio-demographic characteristics and delivery care use.

## Results

### Socio-demographic characteristics of respondents

Information collected on the socio-demographic characteristics of mothers interviewed included their age, level of education, marital status, parity, employment status, place of residence, religion and ethnicity. As shown in Table 1, majority of the mothers were within the age bracket of 20–29 years (58 %) with only 4.4 % in the age bracket of 40–49 years. A significant proportion of the respondents were married or cohabiting (87.7 %), had had some level of education (85.4 %) with those with Middle/JHS level education in the majority (39.6 %). Only 2.4 % had received tertiary level education.

**Table 1:** Percentage distribution of background characteristics of respondents

Background Characteristics	Frequency	Percent
Age Group		
20–29	239	58.0
30–39	155	37.6
40–49	18	4.4
Total	412	100
Highest level of education		
Pre-school	22	5.3
Primary	108	26.2
Middle/JSS/JHS	163	39.6
Secondary/SSS/SHS/Tech/Voc	49	11.9
Higher than secondary	10	2.4
Don’t know	6	1.5
No education	54	13.1
Total	412	100.0
Marital status		
Married or cohabiting	355	87.7
Divorced/separated	14	3.5
Widowed	2	0.5
Never married/never cohabited	34	8.4
Total	405	100
Religious affiliation		
Catholic	42	10.2
Anglican	4	1.0
Methodist	54	13.1
Presbyterian	16	3.9
Pentecostal/charismatics	147	35.7
Other Christian	81	19.7
Moslem	45	10.9
Traditional	4	1.0
Spiritualist	6	1.5
No religion	11	2.7
Other	2	0.5
Total	412	100.0
Employment status		
Unpaid family worker/farmer	60	16
Unemployed	5	1.3
Self-employed	218	58
Employee - formal work (paid)	42	11.2
Informal work (paid)	31	8.2
Others	20	5.3
Total	376	100.0
Parity		
1	125	30.3
2	109	26.5
3	65	15.8
4	35	8.5
5 and above	78	18.9
Total	412	100.0

In terms of ethnicity, 78.4 % of the total respondents were Akans with majority from the Fante ethnic group. The other Akan ethnic groups mentioned were Asante, Akyem and Bono. This finding was not surprising as most residents of the Central Region belong to the Fante ethnic group. Ewes were the second largest ethnic group (16 %) with mothers from other ethnic groups mostly of northern Ghana decent (Nanumba, Dagomba and Hausa) forming 3.9 % of the sample. The mothers were mostly Christians (83.6 %) with the majority (35.7 %) affiliated to Pentecostal or Charismatic churches as is the trend nationally. A greater proportion of the respondents were self-employed (58 %) and engaged mostly in trading activities with only 5.8 % engaged in formal employment. A little over half of the mothers (58.5 %) resided in urban areas with 41.5 % residing in rural localities which also reflect the national trend of increasing urban residence in Ghana. The proportion of Ghanaians living in urban areas has increased from 43.8 % in the year 2000 to 50.9 % in 2010 [16].

### Determinants of delivery service use

Three binary logistic regression models were used to identify the determinants of delivery care use under the fee exemption policy. Chi-square tests were also used to assess the statistical associations between utilization and selected independent variables. More than half (65 %) of mothers interviewed delivered their most recent birth with the policy. Comparing the two districts, utilization of delivery services was rather low for the Assin North Municipal Area 54.4 % (*n* = 112) compared with 75.7 % (*n* = 156) in Cape Coast Metropolis).

From the Chi-square test results, religion (0.000), place of residence (rural/urban) (0.000), parity (0.003) and marital status (0.010) had a statistically significant association with delivery care use. Comparing the two districts, the variables that had a statistically significant relationship with utilization of delivery services in the Cape Coast Metropolis were education, marital Status, religion and parity. Religion and place of residence were significantly related to the use of delivery services in the Assin North municipal area. Maternal age, education, employment status, place of delivery and attendant at delivery did not have a statistically significant relationship with utilization of delivery care.

The results of the three binary logistic regression models are presented in Table 2. Model 1 which contains variables on the socio-demographic characteristics of the woman confirmed parity, religion, marital status and place of residence as significantly related to delivery care use under the free maternal healthcare policy.

**Table 2:** Binary logistic regression results of predictors of delivery care use using individual, husband/partner and health policy factors

Variable	Model 1 Individual variables	Model 2 Individual/Partner variables	Model 3 Individual/Partner/Health Policy variables
	Odds Ratio	Odds Ratio	Odds Ratio
Age Group (RC = 40–49)			
20–29	0.178	0.088**	0.069**
30–39	0.421	0.271	0.247
Parity (RC = 5 and above)			
1	3.405**	4.998**	5.283**
2	3.974***	5.338**	6.390***
3	4.171**	4.675**	5.713**
4	2.146	3.248	3.087
Religion (RC = Muslim)			
Christian (Catholic)	0.719	0.584	0.527
Christian (Orthodox)	0.146***	0.110***	0.077***
Other Christian (SDA, Jehovah’s Witnesses)	0.344*	0.305	0.283
Christian (Pentecostal/Charismatic)	0.581	0.751	0.847
Other (Traditional/Spiritual/No religion)	0.299	0.613	0.636
Education (RC = Other – Vocational, Technical)			
No Education	1.019	0.788	0.968
Primary	1.006	0.450	0.427
Middle/JHS	1.771	1.214	1.141
Secondary/SHS/Technical	2.403	2.343	2.634
Higher than Secondary	3.226	0.263	0.217
Ethnicity (RC = Other (Ga-Adangme, Guan, Hausa))			
Akan	1.531	2.873	2.905
Ewe	2.049	3.151	2.724
Marital Status (RC = Single (Never married, Never-cohabited)			
Married/cohabiting	0.316*	0.124	0.094
Formerly in union (Widowed, Divorced/Sep)	0.114**	0.178	0.098
Residence (RC = Rural)			
Urban	3.566***	3.188***	3.793***
Employment Status (RC = Other – seasonal work)			
Unemployed	2.151	2.969	4.714
Self-employed	1.416	1.448	1.809
Employee (Paid)	0.994	4.691	13.629
Informal work (paid)	2.896	3.891	4.673
Husband/Partner Education (RC = Other- Vocational, Technical)			
No education		0.791	0.652
Primary		4.297	5.177
Middle/JHS		4.740	6.222
Secondary/SHS/Technical		2.336	2.925
Higher than Secondary		7.573	8.317
Husband Employment Status (RC = Other – seasonal work)			
Unpaid Family worker		1.317	1.227
Unemployed		3.075	3.150
Self-employed		1.889	1.784
Employee (Formal work paid)		3.654	6.386
Informal work (paid)		3.096	3.320
Age of husband		1.024	1.022
Awareness about policy (RC = No)			
Awareness about policy (Yes)			13.820*
Full knowledge about policy (RC = No)			
Full Knowledge (Yes)			2.985***
Constant	2.723	0.532	0.020
Nagelkerke R^2^	0.302	0.380	0.433

*Note:* For significance levels

*RC* reference category

*p < 0.05; **p < 0.01; ***p < 0.001

In model 1, at *p* ≤ 0.05, mothers aged 20–29 and 30–39 were 0.178 and 0.421 respectively less likely to use delivery services than those aged 40–49. The likelihood of using delivery care under the policy was lower for mothers who were married or co-habiting and mothers who were either widowed, divorced/separated compared to those who were single. Mothers who resided in urban areas were 3.57 times more likely to use delivery services under the policy than those in rural areas. Mothers with 1, 2 and 3 births had higher odds of delivering with the policy than those with five or more births. Orthodox Christians and other Christians of the Seventh Day Adventist (SDA) group and Jehovah’s Witnesses were less likely to deliver with the policy compared to Muslims. The relationship between mother’s education, ethnicity and employment status and delivery care use was not significant statistically.

Model 2 contained variables on the socio-demographic characteristics of mothers as well as that of their partners. In model 2, all the background characteristics of mothers identified as predictors of delivery service use in model 1 still remained statistically significant except for the marital status of the mother. The variables on husbands’/partners’ characteristics introduced (age, education and employment status) were not statistically significant predictors of delivery service use. The introduction of the husbands’/partners’ variables was however important as it helped to increase the adjusted coefficient of determination (R^2^) from (0.302) in model 1 to (0.380).

Model 3 contained variables on the socio-demographic characteristics of the woman and that of the husband or partner as well as variables on the free delivery policy. The results identified religion, parity, place of residence and maternal age as statistically significant predictors of delivery service use. In addition, awareness and full knowledge about the free maternal healthcare policy were also found to be statistically significant predictors of delivery service use. Maternal education, ethnicity and employment status were not observed as statistically significant predictors of delivery service use under the policy.

The odds of delivering with the policy was 2.985 times higher for mothers who had full knowledge about the policy relative to those who did not have full knowledge about the policy. Similarly, mothers who were aware of the existence of the free maternal healthcare policy were 13.820 times more likely to have delivery services under the policy than those who were not aware of the policy.

The likelihood of using delivery care under the policy was lower for mothers aged 20–29 compared to those in the age bracket of 40–49 (Odds ratio (OR) = 0.069, *p* = 0.003). Mothers who already had 1, 2 or 3 children were more likely to deliver their index child under the policy than those with five or more births. Mothers living in urban areas were 3.793 times more likely to use delivery services under the policy than those living in rural areas (OR = 3.793, *p* = 0.000).

The introduction of variables on the fee exemption policy (awareness and full knowledge of the free delivery’s benefit package) was relevant as those variables were also significant in explaining delivery care use under the policy. Additionally, model 3 was an improvement over models 1 and 2 as the adjusted coefficient of determination (R^2^) increased further to close to 45 % (0.433) compared to 0.302 for model 1 and 0.380 for model 2.

## Discussion

In this study we found that over two-thirds of mothers who participated in the study used supervised delivery services under the fee exemption policy for maternal healthcare for their most recent birth. Utilization was however lower for mothers in the largely rural district than those from urban districts. A little over 7 out of 10 mothers in urban areas delivered for free under the policy compared to approximately 5 in 10 for mothers in rural areas. The findings is in line with an earlier study that has confirmed a possibility of marked variations in utilization of maternal healthcare services under given healthcare financing mechanism due to challenges in the design and/or implementation of the policy or initiative [19].

The study also found the major determinants of delivery care use under the policy as, age, religion, marital status, parity, place of residence, awareness and knowledge about the fee exemption policy.

The likelihood of using delivery care under the policy was lower for younger mothers aged 20–29 compared to older mothers in the age bracket of 40–49. The finding is inconsistent with previous studies [20] and at the same time consistent with others [21, 22]. The relatively lower use of the free delivery services by younger mothers could be partly attributed to concerns of the quality of maternity care received under the policy. The Central region has been particularly noted as one region with a very low uptake of care provided under the National Health Insurance Scheme (NHIS) which includes free maternity care and lack of confidence in the programme has been noted as one major reason [23]. Younger mothers who most likely will be having their first births will most likely be very enthusiastic about receiving the most efficient care and may therefore choose to pay for maternity services which many consider to be of a better quality than services provided for free. Older mothers particularly those who already have many children on the other hand may have varied expenditures to deal with and would therefore not prefer to add that of an additional birth if they can receive care at no cost.

The study also found that mothers with lower parity had higher odds of delivering with the policy than those with higher parity. Previous studies have also confirmed a strong association between lower parity and use of maternal healthcare services [22, 24]. Within the study setting usage of free care was lower for mothers with higher parities relative to those with lower parities partly because ordinarily, women with many children have been exposed to a number of childbearing episodes and therefore will not be too enthusiastic and religious to seek for supervised care even when it comes at no cost.

On religion, the results showed that orthodox Christians and other Christian religious groups as the Seventh Day Adventists (SDA) and Jehovah’s Witnesses had a lower likelihood of delivering with the policy than Muslim women. There was no significant relationship between supervised care use under the policy and religion among mothers who were Traditionalist/Spiritualist. The finding on religion may suggest differences in religious ideologies in seeking for supervised/facility-based services during pregnancy and childbirth among different religious groups. Previous studies [21, 25, 26] have confirmed a strong association between religion and health-seeking behaviours during pregnancy, delivery and the post-partum period.

Place of residence has been found to constitute a major determinant of healthcare use as it shapes individual opportunities and exposure to healthcare resources [22, 27]. It is therefore not surprising that place of residence was highly significant in predicting delivery care use under the policy. Similar to what has been reported in earlier studies [22, 27, 28], mothers residing in rural areas were 3.79 times less likely to use delivery services under the free delivery policy than those living in urban areas. These studies have shown that urban settlements in Ghana and other developing economies receive a better share of healthcare resources including supervised maternity services. Additionally, well-known community-level barriers including poor transportation networks and distance to available healthcare facilities could partly explain variations in access to services between women in rural and urban areas [20].

Most rural communities are disadvantaged when it comes to the distribution of health infrastructure, human and medical resources and the availability of transportation infrastructure to the nearest health facility. The findings suggest the need for increased attention to improving healthcare resources in rural areas to ensure that resources are equitably distributed across rural and urban areas. Mothers will be willing and happy with using supervised care during pregnancy and childbirth if a good facility is available and accessible to them.

The study also introduced variables on health policy in the regression analysis as it has become imperative to look beyond individual- and community-level factors in addressing challenges to accessing supervised care at birth. Addressing challenges associated with accessing care under a given healthcare policy is largely beyond the remit of the individual woman. The variables introduced (awareness and knowledge about the fee exemption policy) were statistically significant in explaining delivery care use under the policy.

Some of the findings of the study were not consistent with certain hypotheses explaining maternal healthcare service use. For instance maternal education which has often been identified as a catalyst to empowering women and exposing them to better use of healthcare services [20, 24, 27] was not statistically associated with delivery care use in the study setting. This could be attributed to the general low level of education of most indigenous inhabitants of the Central region. The region has been classified among the poorest regions in Ghana with majority of its inhabitants not highly educated [23]. Education could therefore not be critical in influencing women’s delivery decisions in this study.

The study acknowledges some limitations which should be considered in the interpretation of the results. First, even though the study was undertaken in a pilot region in which the fee exemption policy was implemented, the findings cannot be generalized for the entire region since only 2 districts out of the nineteen (19) existing districts were selected. Secondly, the findings can best inform decisions regarding effective implementation of the delivery fee exemption policy at only the micro level. Furthermore, the study was cross-sectional and employed the use of a structured questionnaire to collect primary data. Information collected from the questionnaire survey was largely reliable in answering the study’s objective of identifying factors that influences women’s use of supervised care at birth. A questionnaire survey is however limited in so far as it does not allow for probing to understand further and provide meanings to the study’s findings. Even though useful, the present study was not intended to provide qualitative insights into how the factors identified influence women’s use of supervised delivery care under the policy.

## Conclusions

The study identified place of residence (rural/urban) as one of the strongest determinants of supervised care use under the free maternal healthcare policy with mothers residing in rural areas less likely to use the services than those in urban areas. The results on place of residence have very useful implications for effective implementation of the free delivery policy with regards to improving equitable access to care for both rural and urban inhabitants. The finding suggests that efforts at improving access to supervised care under the “free delivery” policy should not only target disadvantaged individuals but also disadvantaged localities in which many poor and disadvantaged women live.

Additionally individual and policy level factors affecting delivery service use such as maternal age, parity, marital status, awareness and knowledge about the free delivery policy have been identified in the study. Service improvement interventions that take cognisance of these factors are likely to have an impact on utilisation of supervised care under the delivery fee exemption policy.

Overall, the findings of this study have policy implications for addressing some unfinished business of Millennium Development Goal (MDG) 5 on improving maternal health under the Sustainable Development Goals (SDGs). In particular, the findings from this study provide useful lessons for realizing targets for improving maternal and child health (targets 3.1, 3.2 and 3.8) under SDG 3 which seeks to ensure healthy lives and promotes well-being for all at all ages. A realistic and achievable inter-sectorial intervention programmes aimed at addressing both individual and policy implementation bottlenecks should be put in place to improve maternal health during the post MDG period especially for rural localities.

## Abbreviations

ADDRF, African Doctoral Dissertation Research Fellowship; APHRC, African Population and Health Research Centre; DAAD, Deutscher Akademischer Austausch Dienst/German Academic Exchange Service; DHMT, District Health Management Team; IDRC, International Development Research Centre; IRB, Institutional Review Board; ISSER, Institute of Statistical Social and Economic Research; MDG, Millennium Development Goal; NHIS, National Health Insurance Scheme; NMIMR, Noguchi Memorial Institute for Medical Research; SDA, Seventh Day Adventist; SDGs, Sustainable Development Goals; SPSS, Statistical Package for the Social Sciences
